# Pragmatic language impairment in children with Noonan syndrome

**DOI:** 10.1080/02699206.2016.1188422

**Published:** 2016-06-27

**Authors:** Magnhild Selås, Wenche Andersen Helland

**Affiliations:** ^a^ Department of Biological and Medical Psychology, Faculty of Psychology, University of Bergen, Bergen, Norway; ^b^ Section of Research & Innovation, Helse Fonna, Haugesund, Norway; ^c^ Department of Speech and Language, Statped Vest, Bergen, Norway

**Keywords:** CCC-2, children, language, language impairment, Noonan syndrome, pragmatics

## Abstract

Noonan syndrome (NS) is a disorder causing symptoms like short stature, characteristic facial features, congenital heart disease, possible mental retardation, and pragmatic difficulties. This study describes the pragmatic skills in NS and discusses the linguistic profile of 17 informants aged 6–15 years, by comparing the participants’ scores on the Children’s Communication Checklist, 2nd edition (CCC-2) (Bishop, 2011), with a group of typically developing children of matching age and gender. Language impairments were common in the NS group. The results show that children and adolescents with NS do not have one coherent pragmatic profile. However, 76.5% of the participants displayed communication impairments, and pragmatic skills were significantly lower than in the control group.

## Introduction

Noonan syndrome (NS) is a congenital disorder caused by germline mutations in genes encoding molecules in the RAS-mitogen-activated protein kinase (MAPK) pathway (Allanson et al. 2010; Cesarini et al. 2009). The RAS-MAPK pathway is involved in the regulation of the cell cycle, including regulation of cell growth and development (Mitin, Rossman, & Der, 2005; Schubbert, Bollag, & Shannon, 2007). The RAS-MAPK mutations produce various syndromes with partly overlapping phenotypes. Typical symptoms for NS are postnatally reduced growth, facial dysmorfism, cardiovascular defects, characteristic facial features, congenital heart disease, skeletal abnormalities and possible developmental delay (Tartaglia, Gelb, & Zenker, 2011). Different known mutations account for 60–80% of the affected individuals (Lee et al., 2011; Roberts, Allanson, Tartaglia, & Gelb, 2013). So far, the underlying mutations in the remaining portion of the affected individuals have not been identified (Cesarini et al., 2009). NS may be inherited or sporadic; it affects males and females equally and the estimated incidence is 1:1000–1:2500 (Mendez & Opitz, 1985) (a ratio 1:1000–1:4000 has also been reported) (Shaw, Kalidas, Crosby, Jeffery, & Patton, 2007).

NS varies in both expressivity and penetration, and there is great phenotypic variation, even within the same mutation (Pierpont, Pierpont et al., 2010). There is a presumed higher rate of persons with a milder expression of the syndrome (Allanson, 2007; Pierpont, Tworog‐Dube, & Roberts, 2013), and the syndrome is probably underdiagnosed (Molven et al., 2009). Romano et al. (2010) describe extremely variable neurologic, cognitive and behavioural symptoms of NS, including tendencies to score poorly on verbal ability tests. Earlier research has shown a larger variability in language abilities in NS groups compared to the typically developing (TD) groups. Communication problems (Wood et al., 1995), low scores on language tests (Lee, Portnoy, Hill, Gillberg, & Patton, 2005) and poorer scores on attention skills and executive functioning than unaffected siblings are reported (Pierpont, Tworog-Dube, & Roberts 2015). However, Pierpont, Weismer et al. (2010) found that the majority of the participants with NS scored within the normal range on general language tests, although a subset of individuals had significant language difficulties. Furthermore, language was significantly correlated with nonverbal cognition, hearing ability, articulation, motor dexterity and phonological memory.

Language form, content and use are essential for communication, and any problems within these areas may cause language impairments (Geurts & Embrechts, 2008). Pragmatics refers to the ability to use and interpret language appropriately in different social contexts (Bishop, 1997) and influences discourse management, communicative intention and presupposition. Children with pragmatic language impairment (PLI) might speak fluently and articulate clearly with well-formed sentences, but they have problems understanding and adapting to the needs of their conversational partners. They might give inappropriate responses, have problems with timing in conversations or misunderstand because they interpret what is said too literally (Helland, Lundervold, Heimann, & Posserud, 2014).

The relationship between PLI and specific language impairments (SLI) has been debated. SLI is a heterogeneous disorder displaying a diversity of linguistic outcomes especially regarding language form. SLI has been defined as a developmental disorder in which children experience significant limitations in language abilities. Their cognitive skills, however, are within normal range, with no neurological, sensory or physical impairments that directly affect the use of spoken language (Bishop & McDonald, 2009). PLI, on the other hand, has been described as a sub-group of SLI, part of the autism spectrum disorder (ASD) or an intermediate condition between SLI and ASD (Bishop, 2003; Norbury, Nash, Baird, & Bishop, 2004). Difficulties in pragmatic functioning might overlap with autistic-like behaviour, and children with NS often have symptoms characteristic of ASD without fully meeting the diagnostic criteria (Adviento et al., 2014; Alfieri et al., 2014; Pierpont, 2015). Alfieri et al. (2014) reported autistic features in 12% of the NS subjects in their study. According to Pierpont (2015), social aspects of language might be affected in NS, and it is not clear whether the prevalence of autistic features is a consequence of intellectual, communicatory or sensory impairments typical for NS. Other explanations of the autistic-like behaviour are deficits in social interaction (reciprocity) or social cognition, or alexithymia (problems talking about emotions) (Roelofs et al., 2015; Verhoeven et al., 2003; Wingbermühle et al., 2012). Pierpont (2015) asks for studies assessing pragmatic language and social communication in individuals with NS.

Pragmatic problems have been reported to be more prevalent in NS than in TD children. According to Pierpont, Weismer et al. (2010), 40% of their participants failed to meet the criterion for an age-appropriate level of pragmatic function. Furthermore, pragmatic problems were significantly more prevalent among boys than girls. This finding relates to Adviento et al. (2014), who report a small male bias in autism-like impairments in NS.

Articulation problems have also been described in NS. Nora et al. report articulation problems in 72% of the subjects in their study (Nora, Nora, Sinha, Spangler, & Lubs, 1974), while Pierpont, Weismer et al. (2010) report 20%. Shah, Rodriguez, St Louis, Lindley, and Milla (1999) and Romano et al. (2010) point to frequent referrals to speech/language therapists and/or feeding specialists due to problems related to the articulatory organs, such as significant feeding, sucking and swallowing problems. According to Pierpont, Weismer et al. (2010), 70% of children with NS receive speech/language therapy. Hearing impairments are also common in NS and might cause articulatory problems (Geelan-Hansen & Anne, 2015; van Trier et al., 2015).

The aims of the present study were to explore whether children with NS can be differentiated from a group of TD children in terms of their language profiles and to investigate PLI in these children. Based on earlier research, we hypothesised (1) that children with NS would score lower on pragmatic abilities than TD children and (2) that the variability would be larger in the NS group than in the TD group. Moreover, we expected (3) that NS children would have more frequent articulation problems than TD children.

## Method

### Participants and procedure

A total of 34 children aged 6–15 years took part in this study: an NS group and a comparison group of TD children. The NS group consisted of 17 children (12 males, 5 females; mean age 10.2 years, SD 37.29 months) and the TD group consisted of 17 children (11 males, 6 females; mean age 10.1 years, SD 36.36 months). The children in the NS group were diagnosed either based on gene analysis or by a specialist. Oral and written information about the study and invitations to participate were distributed to parents at a meeting for children and adolescents with NS at a Norwegian National Health Centre for rare diseases in March 2013. Additionally, information and invitations to participate were presented on the website and Facebook page of the Norwegian Noonan Syndrome Association (‘Noonanforeningen’), and potential participants were contacted personally by email and phone. One parent contacted the first author to participate in response to the information on the NS Association’s website. The parents of the participating children completed the Children’s Communication Checklist Second Edition (CCC-2; Bishop, 2011) and returned it together with a letter of informed consent. To minimise possible misunderstandings, most parents completed the questionnaires in the presence of the first author. A total of 22 questionnaires were handed out, and 19 were completed and returned. Two respondents were excluded because they did not have a confirmed diagnosis of NS, which meant that the study had 17 NS participants.

Seventeen TD children who had previously taken part in a validation study of the Norwegian adaptation of the CCC-2 (Helland, Biringer, Helland & Heimann, 2012) were selected as a comparison group. According to their parents, these children did not have any language problems nor did they have any known learning disabilities or special education needs. The children in the NS group and the children in the TD group were matched by age and gender (one boy in the NS group was matched to a TD girl because age was considered more important than gender). The comparison group thus consisted of 11 males and 6 females.

The study was approved by the Norwegian Regional Ethics Committee for Medical and Health-related Research (REK), University of Bergen, Norway.

### The instrument

The CCC-2 (Bishop, 2003) is a parent-completed checklist, designed to distinguish children with language impairment from TD children and to identify pragmatic and structural language impairments. The Norwegian adaptation of the CCC-2 (Bishop, 2011) has good internal consistency (alpha ranging from 0.73 to 0.89) and inter-rater reliability (ranging from 0.44 to .076) (Helland, Biringer, Helland & Heimann, 2009). The CCC-2 consists of 70 items grouped into 10 subscales: A: speech, B: syntax, C: semantics, D: coherence, E: inappropriate initiation, F: stereotyped language, G: use of context, H: nonverbal communication, I: social relations and J: interests. Each subscale contains seven items, with five items describing difficulties and two items describing strengths. The first eight subscales (A–H) assess structural and pragmatic aspects of language, while scales I and J assess behaviours known to be impaired in cases of ASD. Each item is scored on a 4-point scale, indicating the frequency of the behaviour described: less than once a week (or never), at least once a week, but not every day, once or twice a day and several times a day or always. The raw scores are converted into scaled scores with a mean of 10 and an SD of 3 by an automatic scoring programme that comes with the CCC-2. While a high raw score indicates poor language ability, a high scaled score indicates better language ability. Two composite scores are derived: (1) The General Communication Composite (GCC) is derived by adding the scaled scores of the first eight subscales (A–H). This composite is an overall measure of communication skills and is an effective means of distinguishing children with language impairments from TD children. A GCC below 55 is the cut-off for language impairment. (2) The Social Interaction Deviance Composite (SIDC) is a difference score designed to identify children who show pragmatic impairments disproportionate to their structural language abilities. The SIDC is derived by subtracting the sum of scales A–D (measuring language form) from the sum of scales E, H, I and J (measuring pragmatic competence). If the pragmatic skills are weak but the language structure skills are relatively good, this will produce a negative SIDC. If the pragmatic skills are good but the structural skills are relatively weak, the SIDC will be positive. However, the SIDC is only to be interpreted if the GCC is below cut-off (55), or if the SIDC is −15 or below. A GCC below cut-off and a negative SIDC indicate better structural than pragmatic (and social) abilities, which is a profile typically seen in children with PLI or in children with features of ASD (Bishop, 2003). If the GCC is below cut-off and the SIDC is higher than 9, this indicates better pragmatic than structural abilities, a linguistic profile characterising SLI (Norbury et al., 2004). Additionally, an unstandardised pragmatic composite (PC), although not included in the CCC-2 scoring programme, is reported in several studies (Timler, 2014; Volden & Phillips, 2010). The PC is calculated by adding the scaled scores of the subscales D to H (Helland et al., 2014). No Norwegian norms are available for the PC score. However, since 10 is the average of the scaled scores on each of the five scores in the index, one would expect a pragmatic score of 50 as a putative mean value.

The statistical analyses were run using IBM SPSS version 22. Student’s independent sample *t*-tests were used to test group differences, and Cohen’s *d* was calculated to evaluate effect sizes and the results are interpreted according to general guidelines (0.20 is a small effect, 0.50 is moderate and 0.80 is large). Bonferroni correction was made to counteract the effect of repeated measures. Levene’s test for homogeneity of variances was used to compare the variance in GCC, SIDC and PC and on the subscales on CCC-2 between the two groups.

## Results

Table 1 shows the results from the different subtests in CCC-2.

**Table 1.** CCC-2 scaled scores (a high score indicates better language ability): Means and standard deviations, Levene’s test for equality of variances, *t*-test, and effect size for the NS group and the TD group.^1^

	Groups											
	NS		TD				*t*-test for equality of means					
*n*/gender (male/female)	17 (12/5)		17(11/6)		Levene’s test for equality of variances					95% confidence interval of the difference		
	Mean	SD	Mean	SD	*F*	Significance	*t* (*df*)	*p*	Mean difference	Lower	Upper	Effect size *d*
A. Speech	7.18	3.59	9.82	2.27	7.11	0.01	−2.57 (27)	ns	−2.65	−4.76	−0.53	0.6
B. Syntax	6.71	3.57	9.82	2.56	4.68	0.04	−2.93 (29)	ns	−3.12	−5.29	−0.94	1.0
C. Semantics	4.75	3.07	10.18	2.96	0.12	0.74	−5.17 (31)	ns	−5.43	−7.57	−3.28	1.8
D. Coherence	5.29	3.12	10.88	2.93	0.004	0.95	−5.38 (32)	<.001	−5.59	−7.70	−3.47	1.8
E. Inappropriate initiation	4.88	3.00	10.94	3.09	0.11	0.74	−5.80 (32)	<.001	−6.06	−8.18	−3.93	2.0
F. Stereotyped language	5.82	3.13	10.29	2.71	1.21	0.28	−4.45 (31)	<.001	−4.47	−6.51	−2.43	1.5
G. Use of context	3.53	2.63	9.65	2.50	0.02	0.90	−6.96 (32)	<.001	−6.12	−7.91	−4.33	2.4
H. Nonverbal communication	6.59	2.62	10.12	2.50	0.17	0.68	−4.02 (32)	<.001	−3.53	−5.32	−1.74	1.4
I. Social relations	6.76	3.78	10.65	2.06	3.97	0.06	−3.72 (25)	0.001	−3.88	−6.01	−1.75	1.3
J. Interests	5.06	2.59	10.76	2.80	0.53	0.47	−6.18 (32)	<.001	−5.71	−7.59	−3.82	2.1

^1^Student’s independent sample *t*-test; Bonferroni-corrected *p*=0.005. CCC-2= Children’s Communication Checklist Second Edition; NS = the group with Noonan syndrome; TD= typically developing comparison group; ns = not significant.

Student’s independent sample *t*-test showed that the NS group differed significantly (being more impaired) from the TD group on all subscales of the CCC-2, but after Bonferroni correction was made, significant differences were identified on subscales D–J. In the NS group, the highest score was in the scale assessing pronunciation (speech, scale A), and the lowest scores were in the scales measuring semantics (C), inappropriate initiation (E) and use of context (G). Levene’s test for equality of variances was used to see if the variance on the subscales was the same across groups. On speech (A) and syntax (B), there was a statistically significant difference at the *p* < 0.05 level, while on social relations (J), the results approached significance (*p* = .06). No significant differences were found on the other subscales.

As can be seen in Table 2, the mean GCC score in the NS group was 44.53 (SD 19.93) compared to s 82.18 (SD = 16.75) in the TD group. The two groups differed significantly (*t*(32) = −5.96; p < 0.001). The effect size was large (Cohen’s *d* = 2.05).

**Table 2.** CCC-2 composite scores (a high score indicates better language ability): means and standard deviations, Levene’s test for equality of variances, *t*-test, and effect size for the NS group and the TD group.^1^

	Groups											
	NS		TD				*t*-test for equality of means					
*n*/gender (male/female)	17 (12/5)		17 (11/6)		Levene’s test for equality of variances					95% confidence interval of the difference		
	Mean	*SD*	Mean	*SD*	*F*	Significance	*t* (*df*)	*p*	Mean difference	Lower	Upper	Effect size *d*
General communication composite	44.53	19.93	82.18	16.75	1.24	.27	−5.964(31)	<.001	−37.65	−50.52	−24.77	2.05
Social interaction deviance score	−0.47	10.0	1.59	5.97	3.83	.06	0.73(26)	ns	−2.06	2.06	−7.87	0.25
Pragmatic composite	26.12	13.05	51.88	11.33	.71	.41	6.15(32)	<.001	−25.76	−25.76	−34.30	2.11

^1^Student’s independent sample *t*-test. CCC-2= Children’s Communication Checklist Second Edition; NS = the group with Noonan syndrome; TD= typically developing comparison group.

A total of 13 out of 17 (76.5%) of the children in the NS group were identified as language impaired (GCC below cut-off). Three children (17.6%) in the NS group displayed an SLI profile (GCC below cut-off and a SIDC>9), seven children (41%) displayed a PLI profile (GCC below cut-off and a negative SIDC) and three children showed impaired communication skills without fitting into either the SLI or the PLI profile. Two of the 17 children (11.8%) in the TD group showed impaired communication skills. One child had a GCC of 33 (impaired communication skills) and one had an SIDC of −16, with the GCC within normal limits, indicating poor pragmatic skills.

**Figure 1.**
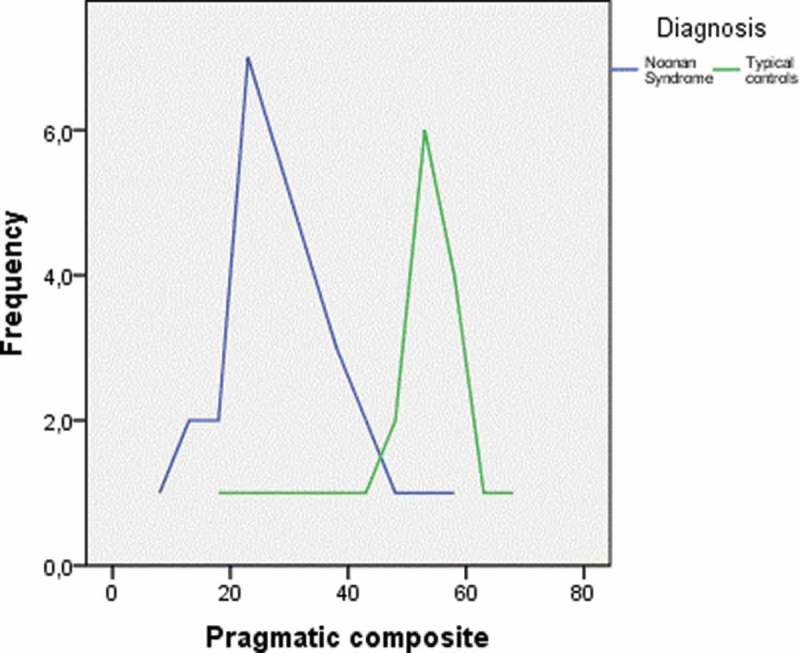
The pragmatic composite scores for the Noonan syndrome (NS) group and the typically developing (TD) controls.

The mean PC score in the NS group was 26.12 (SD = 13.05), compared to 51.88 (SD = 11.33) in the TD group. The children in the NS group scored significantly lower than the children in the TD group (*t*(32) = −6,15, *p* < 0.001). The effect size was large (*d* = 2.1). No difference was found on the SIDC. As shown in Figure 1, there is an overlap in pragmatic skills between the groups, but the majority of NS children score below the TD group.

In this study, the five female participants in the NS group all scored below cut-off on GCC, compared to 8 of the 12 boys. In the TD group, both participants with low scores were boys.

## Discussion

The present study investigated language abilities, focusing especially on pragmatics, in a group of children with NS compared to a group of TD children. Our main findings were that the majority (76.5%) of the children in the NS group was identified with language impairments compared to two children (11.8%) in the TD group. The NS group scored significantly lower than the TD group on an overall measure of communication abilities (GCC) as well as on the PC, thus supporting the view that pragmatics is an aspect of language that poses problems for children with NS.

As Romano et al. (2010) and Pierpont (2015) suggest, NS shows high variability in outcome, including in linguistic performance. We hypothesised that the variability would be larger in the NS group than in the TD group. This has shown to be partly true; no significant differences in the variability were found between the groups in the composite scores GCC and PC. However, on two of the ten subscales, the NS group showed significantly larger variability than the TD group. Earlier studies have found the cognitive skills in NS to vary from high functioning to mild mental retardation (Noonan & Ehmke, 1963; Pierpont, 2015), and verbal performance has shown to correlate closely to general cognitive scores. This is as expected, as clinical groups generally show more variability than TD groups.

In accordance with the findings of Shah et al. (1999) and Romano et al. (2010), we also hypothesised that the NS group would show more frequent articulatory problems than the TD group. Contrary to our expectations, articulation (as measured by subscale A) seems to be of relative strength in the NS group, and the results of the NS group did not differ significantly from the TD group on this subscale.

In this study, all the girls scored in the impaired range on the GCC, compared to 8 of 12 boys. However, the limited number of female participants in this study does not allow for any conclusions on potential gender differences to be drawn.

Pierpont, Pierpont et al. (2010) found that 40% of their participants did not reach an age-appropriate level of pragmatic function. Looking at the individual results of this study, seven children in the NS group showed a PLI profile, three showed an SLI profile, three showed generally weak language skills and four had normal communicative competence. In the TD group, two of the children were identified as language impaired. Our results support earlier findings indicating that in children with NS, pragmatic skills are likely to be impaired. The NS group scored poorly on the scales measuring coherence, appropriate initiation, use of context and interests. This finding is consistent with earlier studies reporting related problems, such as social interaction, social cognition and alexithymia, making it problematic to meet the needs of the conversational partner, make correct inferences and give socially appropriate responses (Roelofs et al., 2015; Verhoeven et al., 2003; Wingbermühle et al., 2012). The results of this study revealed various language profiles in children with NS, including PLI profiles, SLI profiles and generally weak linguistic profiles. Furthermore, a minority has age-appropriate language skills. These findings are consistent with earlier studies reporting varying neurologic, cognitive and behavioural symptoms in individuals with NS (Romano et al., 2010), tendencies to score poorly in verbal ability tests (Lee et al., 2005) and communication problems (Wood et al. 1995). Our finding of impaired pragmatic competence adds to former research on language impairment in children with NS.

## Limitations

The fact that genotype has not been registered may pose a possible limitation to the study. Since it is currently not possible to identify the mutation for all persons with NS (Pierpont, Pierpont et al., 2010), some participants have been diagnosed clinically. In the case of the other participants, the numbers with each mutation were so small that this information was not included. Genotype variation might be able to explain some variation, but the number of participants was too small to enable us to use this information.

NS is probably underdiagnosed (Molven et al., 2009). The participants in this study were recruited through the Norwegian NS Association. It is therefore possible that they have the most severe expression of the symptoms, which may have biased or distorted our findings.

In some cases, NS is autosomal inherited, in others it is due to spontaneous mutations. Linguistic impairment also has a familial factor, implying that some of the parents might have NS themselves and might have problems understanding and answering the questionnaire. This problem was partly solved by the first author being present to answer any questions on most occasions when the forms were filled in.

The fact that this study did not include any assessment of the children’s cognitive abilities may be considered another limitation. However, regarding pragmatics, Bishop and Baird (2001) found that this was not significantly related to intellectual abilities.

In conclusion, our findings underline the importance of assessing language abilities as part of the assessment procedure for children with NS. As children with NS do not constitute a homogenous group, the language profile of each individual child, indicating weaknesses as well as strengths, should be identified. In school, small group instruction, speech and language therapy, modification of assignment loads, the use of multiple modalities in instruction, the spread of learning across multiple sessions and attempts to present information in a meaningful context rather than routine learning have been suggested (Pierpont 2015). Based on the findings of this study, we also suggest giving priority to interventions and treatment plans aimed at improving these children’s pragmatic abilities.
